# The race to develop oral SERDs and other novel estrogen receptor inhibitors: recent clinical trial results and impact on treatment options

**DOI:** 10.1007/s10555-022-10066-y

**Published:** 2022-10-14

**Authors:** Yating Wang, Shou-Ching Tang

**Affiliations:** 1 Ascension Providence Hospital, Southfield, MI USA; 2 Cancer Center and Research Institute, University of Mississippi Medical Center, Guyton Research Building, G-651-07, 2500 North State Street, Jackson, MS 39216 USA

**Keywords:** Selective estrogen receptor degraders, Clinical trials, Protein degradation, Oral SERDs, PROTC, LYTAC, RIBOTAC, Aptamers

## Abstract

Hormonal therapy plays a vital part in the treatment of estrogen receptor–positive (ER +) breast cancer. ER can be activated in a ligand-dependent and independent manner. Currently available ER-targeting agents include selective estrogen receptor modulators (SERMs), selective estrogen receptor degraders (SERDs), and aromatase inhibitors (AIs). Estrogen receptor mutation (ESR1 mutation) is one of the common mechanisms by which breast cancer becomes resistant to additional therapies from SERMs or AIs. These tumors remain sensitive to SERDs such as fulvestrant. Fulvestrant is limited in clinical utilization by its intramuscular formulation and once-monthly injection in large volumes. Oral SERDs are being rapidly developed to replace fulvestrant with the potential of higher efficacy and lower toxicities. Elacestrant is the first oral SERD that went through a randomized phase III trial showing increased efficacy, especially in tumors bearing ESR1 mutation, and good tolerability. Two other oral SERDs recently failed to achieve the primary endpoints of longer progression-free survival (PFS). They targeted tumors previously treated with several lines of prior therapies untested for ESR1 mutation. Initial clinical trial data demonstrated that tumors without the ESR1 mutation are less likely to benefit from the SERDs and may still respond to SERMs or AIs, including tumors previously exposed to hormonal therapy. Testing for ESR1 mutation in ongoing clinical trials and in hormonal therapy for breast cancer is highly recommended. Novel protein degradation technologies such as proteolysis-targeting chimera (PROTACS), molecular glue degrader (MGD), and lysosome-targeting chimeras (LYTACS) may result in more efficient ER degradation, while ribonuclease-targeting chimeras (RIBOTAC) and small interfering RNA (siRNA) may inhibit the production of ER protein.

## Introduction

Breast cancer is the most common malignancy in women. It is the second most common cause of cancer death of women in the USA [1]. Despite an overall 5-year survival rate as high as 89% for early breast cancer, this is reduced to 25% in the presence of metastatic disease [2]. The primary targets of endocrine therapy for breast cancer have been the estrogen receptor alpha (ERa) and/or progesterone receptor (PR). Approximately 70% of breast cancers express ER/PR [3, 4]. Endocrine therapies play an important role in the treatment of HR + metastatic breast cancer (MBC) because of their efficacy and favorable adverse effect profile.

**Fig. 1:**
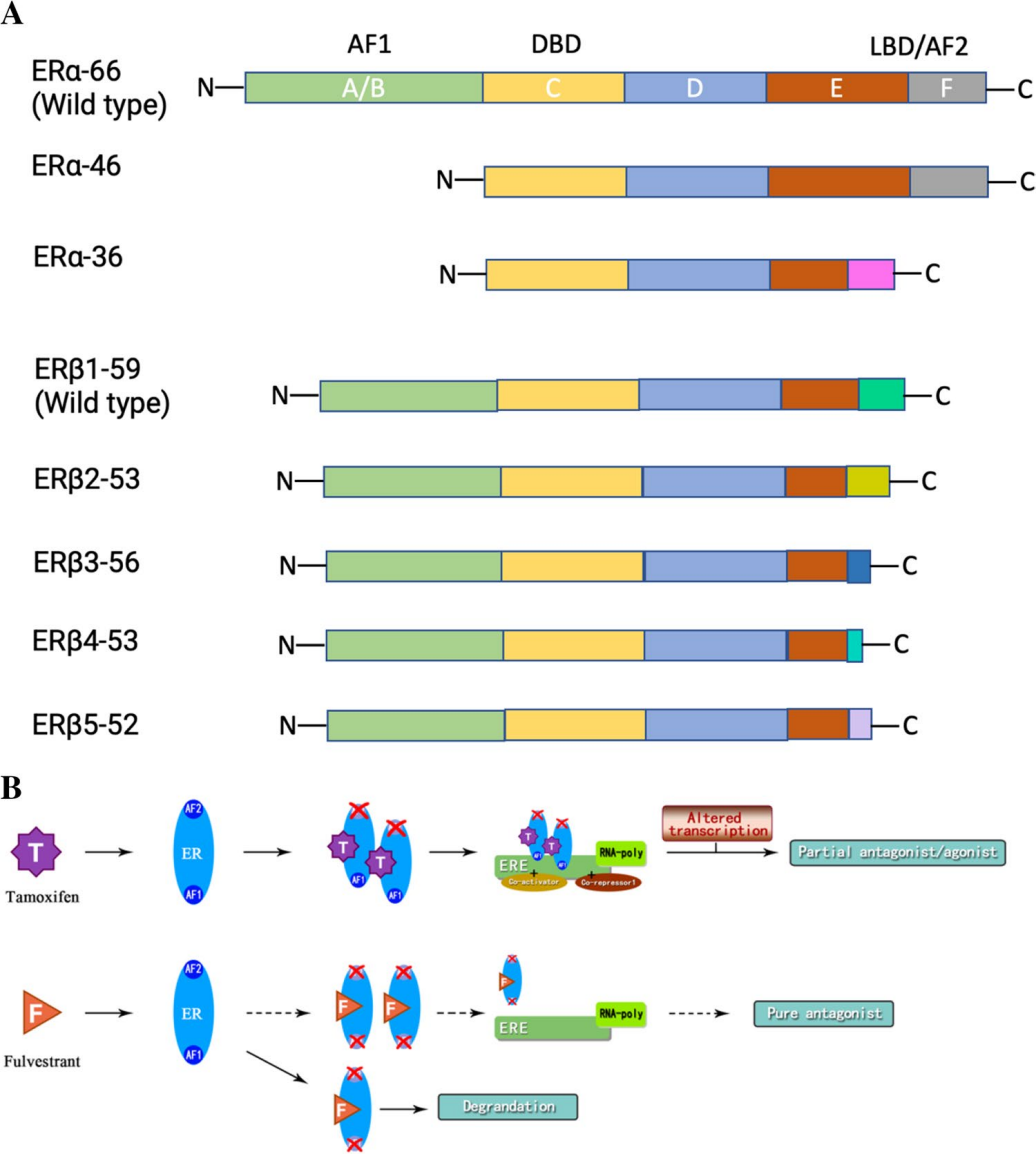
Structure of estrogen receptor isoforms/variants and their interaction with SERM/ SERD. **A** Structure of estrogen receptor (ER) isoforms and their variants. A/B–F: Structural regions. The A/B region contains the amino-terminal domain and the ligand-independent AF-1 (activation function-1) domain; the C region is the binding domain of DNA (DBD); the D region is the hinge region. The D region also encompasses part of the ligand-dependent activation function (AF) domain and the nuclear localization signal. The E and F regions contain the C-terminus region; they encompass the ligand-binding domain (LBD) and ligand-dependent AF-2. The difference of the main isoforms of ERα can be found in region F of ERα36. The ERβ presents variations in the F domain of each isoform. DBD, binding domain of DNA; ER, estrogen receptors; ERα, estrogen receptor alpha; ERβ, estrogen receptor beta; ERα-66, estrogen receptor alpha with molecular weight of 66 kDa; ERβ1-59, estrogen receptor beta with molecular weight of 59 kDa; LBD, ligand-binding domain. **B** Interaction of ER with SERM/SERD. Tamoxifen blocks ERAF1 only as a partial agonist. Fulvestrant blocks both AF1 and AF2 domains as pure antagonist. AF, activation function; ER, estrogen receptor; ERE, estrogen response element; F, fulvestrant; RNA-poly, RNA-polymerase; T, tamoxifen

**Fig. 2:**
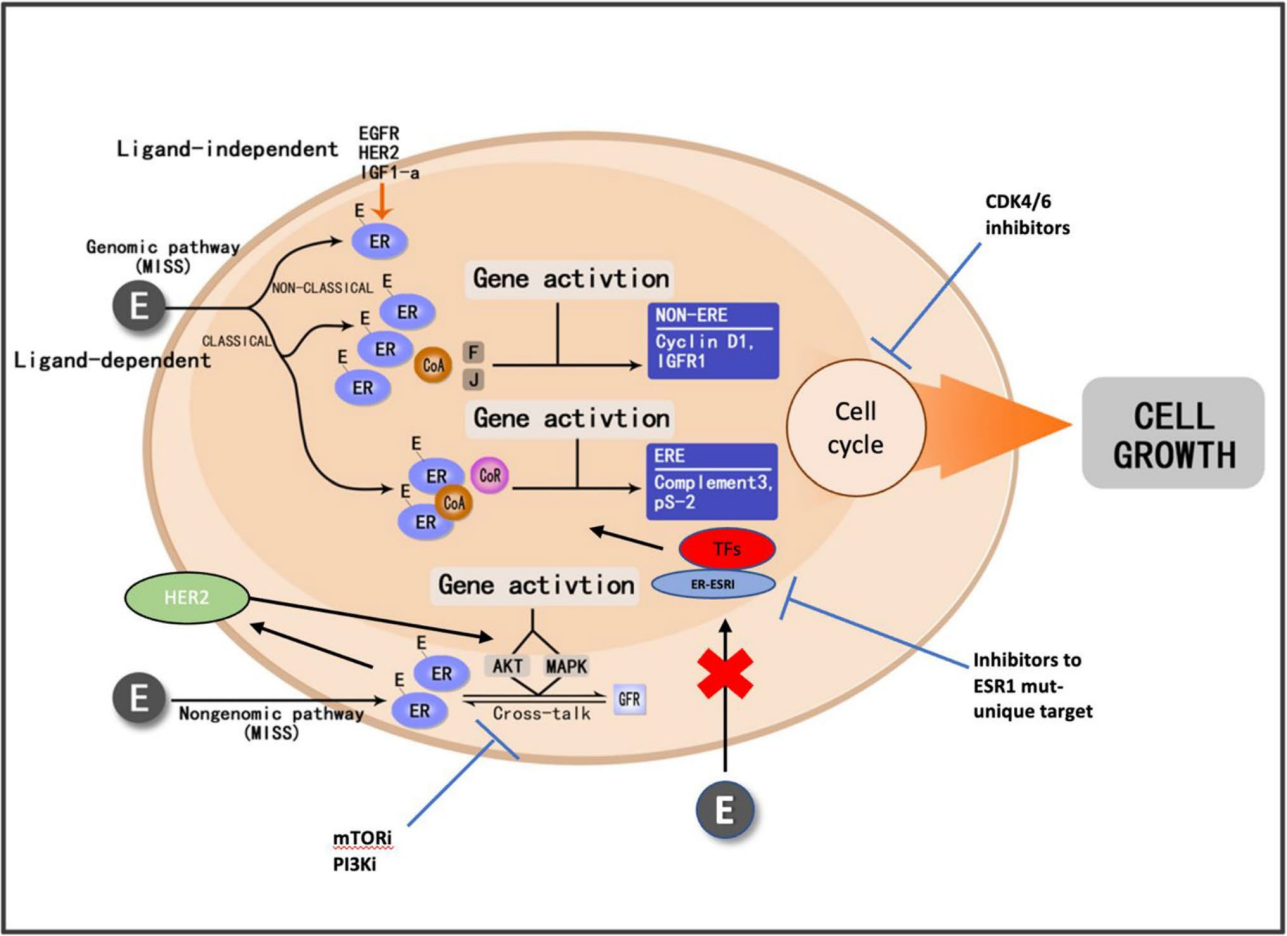
Estrogen receptor signaling pathways: genomic or NISS pathways and the non-genomic or MISS pathway. AKT, protein kinase B; CoA, co-activator; CoR, co-activator receptor; CDK4/6, cyclin-dependent kinase 4/6; ER, estrogen receptor; ERE, estrogen responsive element; FGFR, fibroblast growth factor receptor; HER2, epidermal growth factor receptor 2; IGF1, insulin growth factor 1; MISS, membrane-initiated steroid signaling; mTORi, mammalian target of rapamycin inhibitors; MAPK, mitogen-activated protein kinase; NISS, nuclear-initiated steroid signaling; PI3Ki, phosphoinositide 3-kinase inhibitor; TFs, transcription factors

ER isoforms and their variants are translated from the common mRNA due to different mRNA splicing mechanisms (Fig. 1A) [5]. The regulation of transcriptional activity of estrogen hormone depends on two activating functional domains: AF1 and AF2. The N-terminal AF1 becomes activated independently of estrogen through phosphorylation. Activation of the ligand-binding domain AF2 requires estrogen [6] (Fig. 1B). ERs exist in a dynamic equilibrium between the plasma membrane, cytoplasm, and nucleus [7]. They can translocate from the cytoplasm to the nucleus in hormone-stimulated cells (Fig. 2). As ligand-dependent transcription factors, ER binds to estrogen with its ligand-binding domains (genomic pathways or membrane-initiated steroid signaling), resulting in either direct binding of the ER to estrogen response elements (ERE) in the promoter of target genes (classical pathways) or a protein–protein interaction with other transcription factors at their respective promoter sites (non-classical pathways) to activate or suppress the gene expression (estrogen-dependent pathways) [8, 9]. Additionally, ERs can also act independently of ligands to regulate cell growth (nuclear-initiated steroid signaling), to interact with other growth factor receptors (estrogen-independent pathways) [10] (Fig. 2). More than two-thirds of breast cancers express the ER-alpha protein. Systemic endocrine therapy is considered the oldest and most effective form of targeted breast cancer treatment, which is well-tolerated.

There are three major classes of endocrine therapy drugs with different mechanisms of action used to treat and/or prevent ER + breast cancers, i.e., selective estrogen receptor modulators (SERMs), aromatase inhibitors (AIs), and selective estrogen receptor down-regulators or degraders (SERDs). Among the SERMs available are tamoxifen, raloxifene, and toremifene [11], which act as competitive estrogen antagonists. The most commonly used SERM, tamoxifen, can act as either an antagonist or an agonist depending on the different tissues: despite being an antagonist in breast tissue, it is also an agonist in the uterus and increases the risk of endometrial cancer [12]. Raloxifene, by contrast, does not increase the risk of uterine cancer. Additionally, it is less likely to cause thromboembolic events, cataracts, or other complications but has a higher risk of non-invasive (in situ) breast cancer compared to tamoxifen in early breast cancer [13]. AIs prevent the synthesis of estrogen by inhibiting aromatase. Non-steroidal AIs such as anastrozole and letrozole inhibit aromatase reversibly, while steroidal AIs such as exemestane inhibit aromatase irreversibly. Clinical data indicates that AIs are more effective in preventing disease relapse compared to tamoxifen. While AIs are well-tolerated among patients, their adherence and duration of treatment can be limited by short-term and long-term side effects such as hot flashes, night sweats, menopause, arthralgia/myalgia, and osteoporosis [14].

**Fig. 3:**
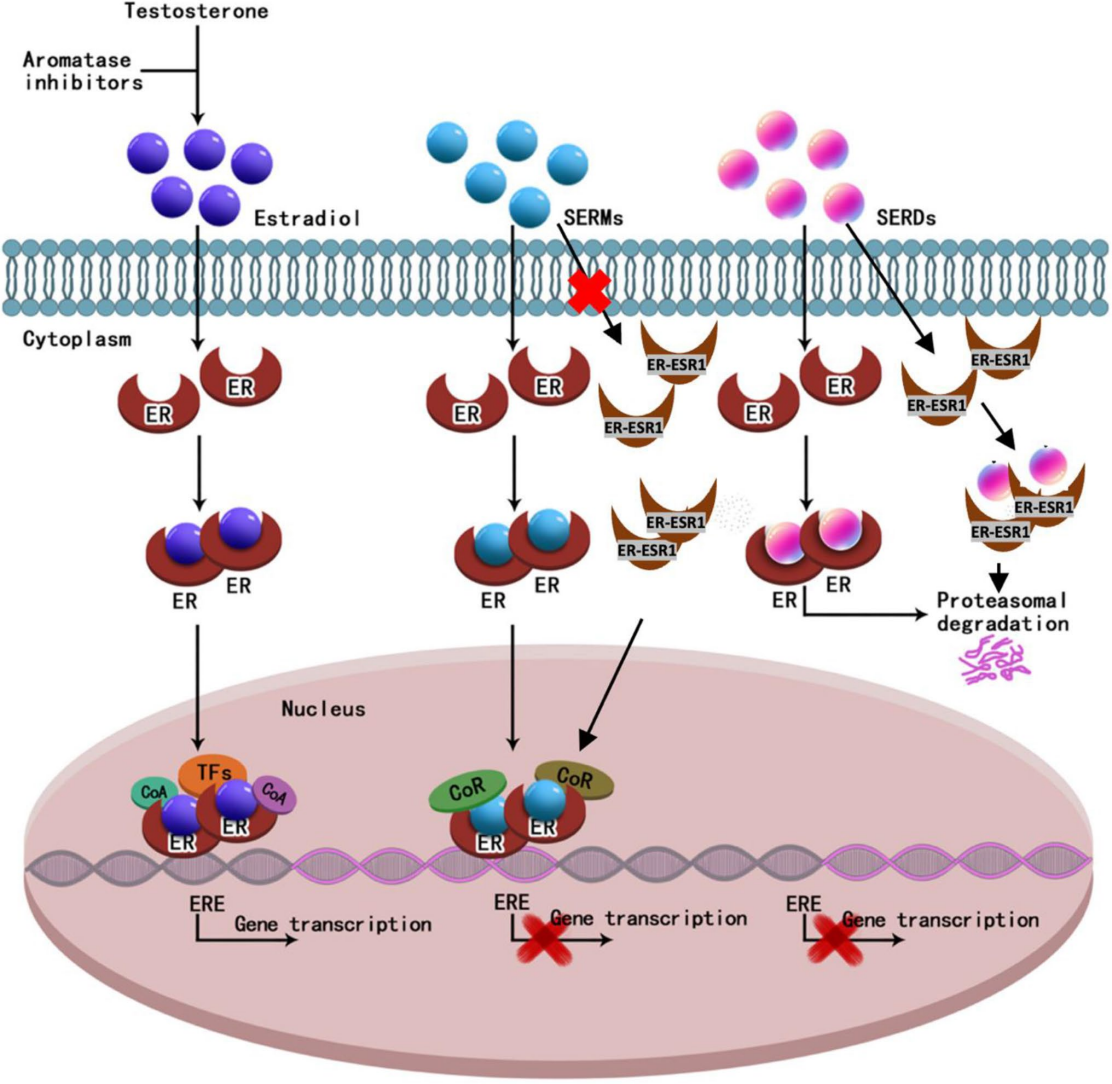
Mechanism of action for estradiol, SERMs, and SERDs. CoA, co-activator; CoR, co-activator receptor; ER, estrogen receptor; ESR1, estrogen receptor 1; ERE, estrogen response element; SERDs, selective estrogen receptor degrader or down-regulator; SERMs, selective estrogen modulators; TFs, transcription factors

SERD binds to ER and induces its degradation (Fig. 3) [15]. Fulvestrant (Faslodex) was introduced as the only marketed SERD in 2002. The binding of ERα by fulvestrant induces a structural change that leads to increased surface hydrophobicity, which attracts the E3 ubiquitin ligase and proteasome causing subsequent degradation [16, 17]. Unlike its SERM counterparts, fulvestrant is a pure ER antagonist with no known agonistic properties. Due to its lack of ligand-dependent effects, fulvestrant inhibits ER ligand-independent functions as well. It is less likely to cause endometrial cancer and thrombosis than tamoxifen [18, 19, 20]. Fulvestrant has been shown to be effective as both first-[21] and second-line therapy for metastatic breast cancer (MBC) [22]. The phase III CONFIRM trial demonstrated a statistically significant increase in both progression-free survival (PFS) and overall survival (OS) at higher fulvestrant doses of 500 mg without increased toxicity, compared to the initially approved 250 mg dose. The randomized phase III FALCON trial demonstrated superior efficacy in disease-free survival (DFS) in favor of 500 mg of fulvestrant compared to anastrozole in the first-line treatment of ER + metastatic breast cancer [23], consistent with the earlier FIRST randomized phase II study that suggested the superiority of fulvestrant over anastrozole in time to tumor progression [24]. Fulvestrant also has increased efficacy in combination with cyclin-dependent kinase (CDK) 4/6 inhibitors such as palbociclib (PALLOMA03) [25], and abemaciclib (MONARCH 2 trial) [26] as the second-line therapy, and ribociclib (MONALEESA-3) [27] as the first- or second-line therapy in metastatic ER + breast cancer compared to fulvestrant alone. In addition, fulvestrant in combination with alpelicib demonstrated superior efficacy compared to fulvestrant alone in tumors with PI3K mutations that had progressed after prior endocrine therapy (SOLAR-1 trial) [28]. Currently, fulvestrant is used as a single agent for low-risk patients or in combination with CDK4/6 inhibitors in the first-line setting, and more commonly alone or in combination with alpelicib in the second-line treatment of ER + MBC.

Due to its low oral bioavailability [29, 30], 250 mg fulvestrant is suspended in 2.5 cc of castor oil and administered as a bilateral intramuscular injection monthly (500 mg). Pain at the injection sites is among the most common complaints among patients, limiting its use in adjuvant settings when 5–10 years of hormonal therapy may be required. In addition, monthly injection caused a large peak and long trough in *in vivo* drug concentration, resulting in suboptimal ER degradation. In the past 15 years, significant efforts were carried out to develop the newer generations of SERDs with superior bioavailability and pharmacokinetics and increased antiestrogenic activity. These have resulted in the discovery of a growing number of oral SERDs, many of which demonstrated robust preclinical and clinical activity to degrade ER and suppress ER + breast cancer. Several of the oral SERDs are undergoing phase III clinical trials to compare with fulvestrant either as a single agent or in combination with other agents such as CDK4/6 inhibitors. Elacestrant is the first oral SERD that has won the race in phase III trials among other competing drugs. In addition, other novel technologies are being explored to degrade ER or inhibit ER production, mostly in preclinical experiments. Here, we summarize the key clinical studies of oral SERDs, ongoing clinical trials, and a glimpse of novel technologies to suppress ER function, aiming to provide an overview of the past, present, and future of oral SERDs and ER inhibitors in the treatment of breast cancer.

## Recent development of oral SERDs

The need to improve the efficacy and its intramuscular formulation resulted in the recent rapid growth in oral SERD development, especially with the emerging role ER ESR1 mutation plays in the resistance to SERM and AIs. In addition, oral SERDs have been shown to not only target ER with ESR1 mutation but enhance the efficacy of agents that target other molecules involved in cross-talks with ER pathways.

PI3K/Akt/mTOR and CDK 4/6/RB pathways have been extensively explored in endocrine resistance in breast cancer. PIK3CA encodes phosphatidylinositol-4, 5-bisphosphate 3-kinase. Its catalytic subunit α is frequently mutated in breast cancer. The key negative regulator of the PI3K/Akt/mTOR pathway is PTEN, which is frequently lost during endocrine therapy as well as Akt and mTOR amplification and/or mutation. ER + breast cancer frequently demonstrates gain of CCND 1 (cyclin D1) and CDK4 and loss of CDKN2A (p16) and CDKN2C (p18). There is a strong relationship between estrogen activity, CDK4/6/RB pathways, and PIK3/Akt/mTOR pathways. Upregulation of cyclin D1 expression and RB phosphorylation and activation of the mTOR signaling pathway have been associated with resistance in ER + breast cancer. Synergistic effects of mTOR inhibitors and endocrine therapy have been demonstrated in clinical trials [31]. BOLERO-2 [32] and TAMRAD [33] trials have demonstrated that the addition of the mTOR inhibitor everolimus to endocrine therapy can reverse endocrine resistance in ER + metastatic breast cancer. Preclinical experiments and ample clinical trials have shown the synergistic effects of targeting CDK4/6/RB pathways in conjunction with endocrine therapy to reduce primary and secondary resistance [34, 35]. Finally, numerous *in vitro* and *in vivo* studies have shown the cross-talk between HER2 and ER and/or progesterone receptor (PgR) pathways. Non-nuclear ER interacts directly or indirectly (e.g., via G protein) with HER2 and activates Ras-MAPK and PI3K-Akt pathways, which results in phosphorylation of ER and other transcription factors (TFs) and coactivators/corepressors (CoA/R), which in turn alters gene expression. There is strong preclinical and clinical evidence showing synergy to combine hormone therapy (HT) and anti-HER2 therapy in HR + /HER + BC [36], as demonstrated in Fig. 2.

The current common treatment approaches for ER/PR-positive MBC involve the combination of AIs and CDK4/6 inhibitors in the first line, fulvestrant alone or with alpelicib in tumors with PI3K mutation in the second line, and everolimus with exemestane, tamoxifen, or single-agent chemotherapy in the third line and beyond.

Elacestrant (GS2-02) is an oral SERD, validated by *in vitro*, *in vivo*, and phase I clinical trials regarding its efficacy in ER degradation and growth suppression of ER + breast cancer [37, 38, 39]. The EMERALD trial (NCT0377893b1) was a multicenter, international, open-label, phase III randomized controlled trial that evaluated elacestrant as monotherapy versus standard of care (SOC) endocrine therapy for the treatment of ER + /HER2-advanced or MBC. The study enrolled 477 patients who had received prior treatment with one or two lines of endocrine therapy, including a CDK 4/6 inhibitor. Both primary endpoints were met, demonstrating a statistically significant and clinically meaningful extension of PFS as monotherapy vs SOC endocrine therapy in the overall population and in the mESR1 population [40]. In the overall population, elacestrant reduced the risk of progression or death by 30% vs SOC. In the mESR1 population, elacestrant reduced the risk of progression or death by 45% vs SOC. PFS rate at 12 months with elacestrant was 22.32% versus 9.42% with SOC in the overall population, and 26.76% versus 8.19% in the mESR1 population. In an analysis excluding the six patients who had received prior fulvestrant and received fulvestrant during the trial, results remained significant in favor of elacestrant, both in the overall population or ESR1 mutation cohort, in terms of statistical significance (*P* = 0.0019; 0.0006), estimates of median PFS (2.8 months vs 1.9 months; 3.8 months vs 1.9 months), 6-month PFS rate (34.3% vs 20.6%; 40.8% vs 19.3%), 12-month PFS rate (22.3% vs 9.5%; 26.8% vs 8.3%), or other efficacy outcomes. A prespecified interim OS analysis planned at the time of the final PFS analysis revealed a trend in favor of elacestrant in all patients (hazard ratio 0.751, 95% confidence interval 0.542–1.038, *P* = 0.0821), as well as significant improvement with elacestrant in patients with ESR1 mutations (hazard ratio 0.592, 95% confidence interval 0.361–0.958, *P* = 0.0325). The final OS analysis is expected in 2023. The most common adverse effects (AEs) observed with elacestrant versus SOC therapy, respectively, included nausea (35.0% vs 18.8%), fatigue (19.0% vs 18.8%), vomiting (19.0% vs 8.3%), decreased appetite (14.8% vs 9.2%), and arthralgia (14.3% vs 16.2%). Grade 3/4 AEs occurred in 64 patients (27.0%) receiving elacestrant and 47 patients (20.5%) receiving SOC therapy. With these results, elacestrant became the first oral SERD to demonstrate higher efficacy than fulvestrant in a pivotal trial in the second-line treatment of MBC. It is expected to become the standard of care in this patient population soon.

Amcenestrant (SAR439859) is another oral SERD that has been validated by preclinical [41] and early clinical trials in ER + breast cancer. AMEERA-3 is an open-label randomized phase II study of amcenestrant (SAR439859) versus treatment of physician’s choice (fulvestrant/aromatase inhibitors/estrogen receptor modular) in locally advanced or metastatic ER + breast cancer. Recently it was reported that the trial had failed on its primary endpoints (PFS) [42]. 79% of patients in the trial received prior CDK4/6i for advanced breast cancer. 90% of patients received fulvestrant in the treatment of physician’s choice arm. PFS was numerically similar between two arms (median PFS 3.6 vs 3.7 months; HR = 1.051 [95% CI: 0.789, 1.4]; *P* = 0.6437). Immature data showed a numerically similar OS. Common (≥ 5% in either arm) treatment-related adverse events (TRAEs) with amcenestrant vs control arm were mostly grade 1/2: nausea (14.0% vs 4.1%), vomiting (8.4% vs 1.4%), hot flush (8.4% vs 7.5%), asthenia (7.0% vs 1.4%), fatigue (5.6% vs 6.1%), and injection site pain (0% vs 6.8%); 4.9% vs 0.7% of patients had grade ≥ 3 toxicities. The phase III AMEERA-5 trial (NCT04478266) was designed to determine whether the addition of amcenestrant to palbociclib would improve PFS vs letrozole plus palbociclib in patients with ER + , HER2 − advanced breast cancer who have not previously received systemic therapy. However, it was recently reported by the sponsor that the study also failed to reach the primary end point of improved PFS. An Independent Data Monitoring Committee (IDMC) found that amcenestrant in combination with palbociclib did not meet the prespecified boundary for continuation in comparison with the control arm and recommended stopping the trial. No new safety signals were observed. Amcenestrant is still currently investigated in the adjuvant setting for patients with ER + breast cancer in collaboration with the Breast International Group (BIG), the European Organization for Research and Treatment of Cancer (EORTC), and Alliance Foundation Trials (AFT). Additionally, the phase III AMEERA-6 trial (NCT05128773) evaluates the safety and efficacy of amcenestrant vs tamoxifen in patients with hormone receptor–positive early breast cancer who discontinued adjuvant AIs because of treatment-related toxicity. However, due to the negative results of AMEERA-3 and AMEERA-5, the sponsor of the amcenestrant recently decided to discontinue the global clinical development program of amcenestrant.

Camizestrant (AZD9833) has been studied in preclinical [43, 44] and early clinical trials [45] as well. In SERENA-6, a randomized, multicenter, double-blind, phase III study, camizestrant or aromatase inhibitor (anastrozole or letrozole) is combined with a CDK4/6 inhibitor (palbociclib or abemaciclib) for the treatment of HR + , HER2-negative MBC with detectable ESR1 mutation. The goal of the study was to evaluate the superiority of AZD9833 over anastrozole or letrozole when combined with palbociclib or abemaciclib. Its estimated primary completion date is September 28, 2023. SERENA-4 is another phase III comparative study that is intended to demonstrate that AZD9833 in combination with palbociclib is superior to anastrozole and palbociclib as the initial treatment of patients with hormone receptor–positive (HR +), human epidermal growth factor 2-negative (HER2 −) advanced/MBC. Its estimated primary completion date is November 10, 2025.

Giredestrant, an orally available SERD, has been investigated in several preclinical [46] and clinical trials [47]. The acelERA is a randomized phase II, open-label multicenter study, which compared the safety and efficacy of giredestrant versus endocrine monotherapy (fulvestrant or aromatase inhibitor) for ER + /HER2 − locally advanced or MBC among males or postmenopausal or pre/perimenopausal females that were previously treated with one or two lines of systemic therapy, one of which had to be endocrine therapy. It was however recently reported that the study’s primary outcome of PFS was not met [47]. The hazard ratio for investigator-assessed PFS (primary end point) was 0.81 with a stratified log-rank *P* value of 0.18. A higher response rate and clinical benefit rate were observed with giredestrant. In patients with *ESR1* mutations, the PFS benefit was more pronounced. Giredestrant was well-tolerated, with a safety profile comparable to control arm. The ongoing persevERA trial, a phase III, randomized, double-blind, multicenter trial, aims to evaluate the efficacy of first-line giredestrant plus palbociclib versus letrozole plus palbociclib among patients with ER + /HER2-negative locally advanced or metastatic breast cancer, whereas the lidERA trial randomized patients with ER + /HER2 − early breast cancer to giredestrant versus endocrine therapy in the adjuvant setting. Both trials are actively enrolling patients as of now.

Imlunestrant (Ly3484356), an oral SERD, has been validated in earlier preclinical [48] and clinical trials [49] in inhibiting ER + breast cancer. EMBER-3 is a phase III, randomized, open-label study of imlunestrant, investigator’s choice of endocrine therapy, and imlunestrant plus abemaciclib in patients with ER + /HER − locally advanced or metastatic breast cancer previously treated with endocrine therapy. The study started on October 4, 2021, and the estimated primary completion date is June 13, 2023.

GDC-0810 (brilanestrant) is a non-steroidal oral SERD. In breast cancer xenograft models with activating mutations in the ESR1, as well as tamoxifen-sensitive and tamoxifen-resistant breast cancer xenografts, GDC-0810 demonstrates potent anti-tumor activity [50, 51]. Despite the ESR1 mutations, GDC-0810 was shown to degrade its target in a preclinical study [52]. An ongoing phase I study is currently in progress investigating its potential to treat advanced ER + breast cancer in postmenopausal women [53].

D-0502 is another oral SERD that is undergoing phase I studies with D-0502 as a single agent and in combination with a standard dose of palbociclib to assess its safety and tolerability. The aim was to identify the maximum tolerated dose (MTD) and/or recommended phase II dose (RP2D), assess the PK properties, and evaluate preliminary anti-tumor activities in women with advanced or metastatic ER + , HER2 − breast cancer. The primary outcome measure is the incidence of AEs meeting protocol-defined dose-limiting toxicity (DLT) criteria.

Additional oral SERDs under development in different stages of clinical trials are listed in Table 1, including the drug name, companies’ name, phase in the clinical trial, primary outcome, and status. The efficacy and toxicities of most common oral SERDs are summarized in Table 2.

**Table 1:** Completed and ongoing clinical trials of oral selective estrogen receptor degraders (SERDs)

Drug name	Study drugs	Trial name/NCT No	Companies	Phase	Primary outcome	Status
Elacestrant (GS2-02 and RAD 1901)	Elacestrant, fulvestrant, anastrozole, letrozole, exemestane	EMERALD	Radius	Phase III	PFS	Active, not recruiting
Amcenestrant (SAR439859)	Amcenestrant, fulvestrant, anastrozole, letrozole, exemestane, tamoxifen	AMEERA-3	Sanofi	Phase II	PFS	Completed
Amcenestrant (SAR439859)	SAR439859, palbociclib letrozole, goserelin	AMEERA-5	Sanofi	Phase III	PFS	Completed
Amcenestrant (SAR439859)	Amcenestrant, tamoxifen	AMEERA-6	Sanofi	Phase III	Invasive breast cancer–free survival (IBCFS)	Discontinued
Camizestrant (AZD9833)	AZD9833 + CDK4/6 inhibitor	SERENA-6	AstraZeneca	Phase III	PFS	Recruiting
Camizestrant (AZD9833)	AZD9833 plus palbociclib versus anastrozole plus palbociclib	SERENA-4	AstraZeneca	Phase III	PFS	Recruiting
Giredestrant (GDC9545)	Giredestrant	NCT04961996	Hoffmann-La Roche	Phase III	Invasive disease-free survival (IDFS)	Recruiting
Giredestrant (GDC9545)	Giredestrant, letrozole, palbociclib, LHRH agonist	NCT04546009	Hoffmann-La Roche	Phase III	PFS	Recruiting
Giredestrant (GDC9545)	Giredestrant, fulvestrant or an aromatase inhibitor (physician’s choice), LHRH agonist	acelERA	Roche	Phase II	PFS	Completed
Imlunestrant (ly3484356)	LY3484356 exemestane fulvestrant	EMBER-3	Eli Lilly	Phase III	PFS	Not yet recruiting
Rintodestrant (G1T148)	G1T48 palbociclib	NCT03455270	G1 Therapeutics, Inc	Phase I	Dose-limiting toxicity (DLT)	Active, not recruiting
SHR9549	SHR9549	NCT03596658	Jiangsu HengRui Medicine Co., Ltd	Phase I	Dose limited toxicity (DLT)	Unknown
ZN-c5	ZN-c5 palbociclib	NCT03560531	Zeno Alpha	Phase I Phase II	Determine a maximum tolerated dose (MTD) or recommended phase II dose (RP2D) for ZN-c5 as a monotherapy	Recruiting
D-0502	D-0502 and in combination with palbociclib	NCT03471663	InventisBio	Phase I	Incidence of AEs meeting protocol-defined dose-limiting toxicities (DLTs) criteria	Recruiting

*CDK4/6*, cyclin-dependent kinase 4/6; *SERD*, selective estrogen receptor degrader; *NCT*, national clinical trial; *PFS*, progression-free survival

**Table 2:** Efficacy and toxicities of oral selective estrogen receptor degraders (SERDs) in development

Drug candidate	Clinical benefit rate	Mean degradation in patient	Select treatment-related adverse event (5% of patients)					CDK4/6i pretreated patients (0–100%)
			Gastrointestinal (GI) AEs			Other AEs		
			Diarrhea	Nausea	Vomiting	Bradycardia	Visual disturbance	
Elacestrant	42.6%	Not reported		√	√			100%
H38-65,545	34%	Not reported	√	√	√	√		87%
ZN-C5	40%	Not reported	√	√	√			87%
Rintodestrant	30%	28%	√	√	√			70%
SAR439859	34%	Not reported	√	√	√			63%
AZD9833	35%	< 50%		√	√	√	√	62%
GDC9545	41%	< 50%	√	√		√		59%

*AEs*, adverse effect; *CDK 4/6i*, cyclin-dependent kinase 4/6 inhibitor; *SERDs*, selective estrogen receptor degraders

## Future development of estrogen degraders

Fulvestrant most likely represents the first targeted therapy in cancer that utilizes the cellular protein degradation system proteasome to inhibit a target or biomarker. In the past decades, many novel technologies and drugs have emerged that utilize different protein degradation mechanisms such as the lysosome system. In addition, actively targeted protein degradation uses bispecific linker or molecular glues to bring together the protein degradation targets, and the degradation systems are being developed. These technologies can target proteins that are not druggable in the past, including secretary proteins.

### PROTAC

PROTAC employs bifunctional molecules; one end binds to a target protein and the other hijacks cellular quality control mechanisms to cause a protein to degrade. Bifunctional PROTAC molecules are composed of a ligand (typically a small molecule inhibitor) that binds to a protein of interest (POI) and a covalently linked ligand of an E3 ubiquitin ligase (E3). A PROTAC binds to POIs and recruits E3 to ubiquitinate them, leading to POI degradation by the proteasome [54]. Over 50 PROTACs have been successfully developed including the ER-targeting PROTAC [55].

AVR-471 is a novel PROTAC ER degrader. A phase I clinical trial found 90% ER degradation by AVR-471, compared to 50% with fulvestrant. AVR-471 remains effective in tumors that had progressed on prior fulvestrant therapy and even oral SERDs. Analysis of 12 paired biopsies from patients treated at 30 to 360 mg daily demonstrated up to 90% ER degradation in tumors expressing wild-type or mutant ER. Of 34 patients who were evaluable for clinical benefit (confirmed complete response, partial response, or stable disease ≥ 24 weeks), the clinical benefit rate was 41%. Among twenty-one patients who had failed five prior lines of treatment, including CDKi and fulvestrant, three patients achieved partial remission, and one patient achieved stable disease with more than 50% shrinkage of target lesions. The most common (≥ 10%) treatment-related adverse events (TRAEs) were nausea (24%), fatigue (12%), and vomiting (10%) which were predominantly grade 1 in severity. Two patients experienced grade 3 adverse events (AEs) that were potentially related to ARV-471. There were no AEs > grade 3 potentially related to ARV-471. All AEs were manageable with only one patient discontinuing ARV-471 due to a TRAE (grade 3 thromboembolism) [56]. Given its promising safety profile, ARV-471 is currently studied in phase I dose escalation studies, a phase Ib combination study with IBRANCE (palbociclib), and a phase II monotherapy dose-expansion study for metastatic breast cancer. Additional phase III trials are expected to be conducted for MBC. C4891001 (2L/3L) will be comparing ARV-471 monotherapy with fulvestrant in patients with ER + /HER2 − unresectable loco-regional recurrent or metastatic breast cancer patients who progressed after based on treatments for advanced disease. C4891002 (1L) is a double-blind study that will be conducted in ER + /HER2 − unresectable loco-regional advanced or metastatic breast cancer patients who have received no prior therapy in an advanced setting comparing ARV-471 plus palbociclib with letrozole plus palbociclib.

### MGD

Not all target proteins possess the sockets (or degrons) to which PROTAC linkers can bind (non-druggable). Molecular glue degrader bypasses this requirement. A “molecular glue” is generally a small molecule that induces or enhances protein–protein interactions (PPI) by forming ternary interactions between proteins that do not normally interact. This results in ubiquitination and subsequent degradation of the target protein. The molecular glue becomes dissociated afterward. As such, these molecular glues possess a catalytic function and can deliver significantly greater target degradation than simply target binding  [57].

**Fig. 4:**
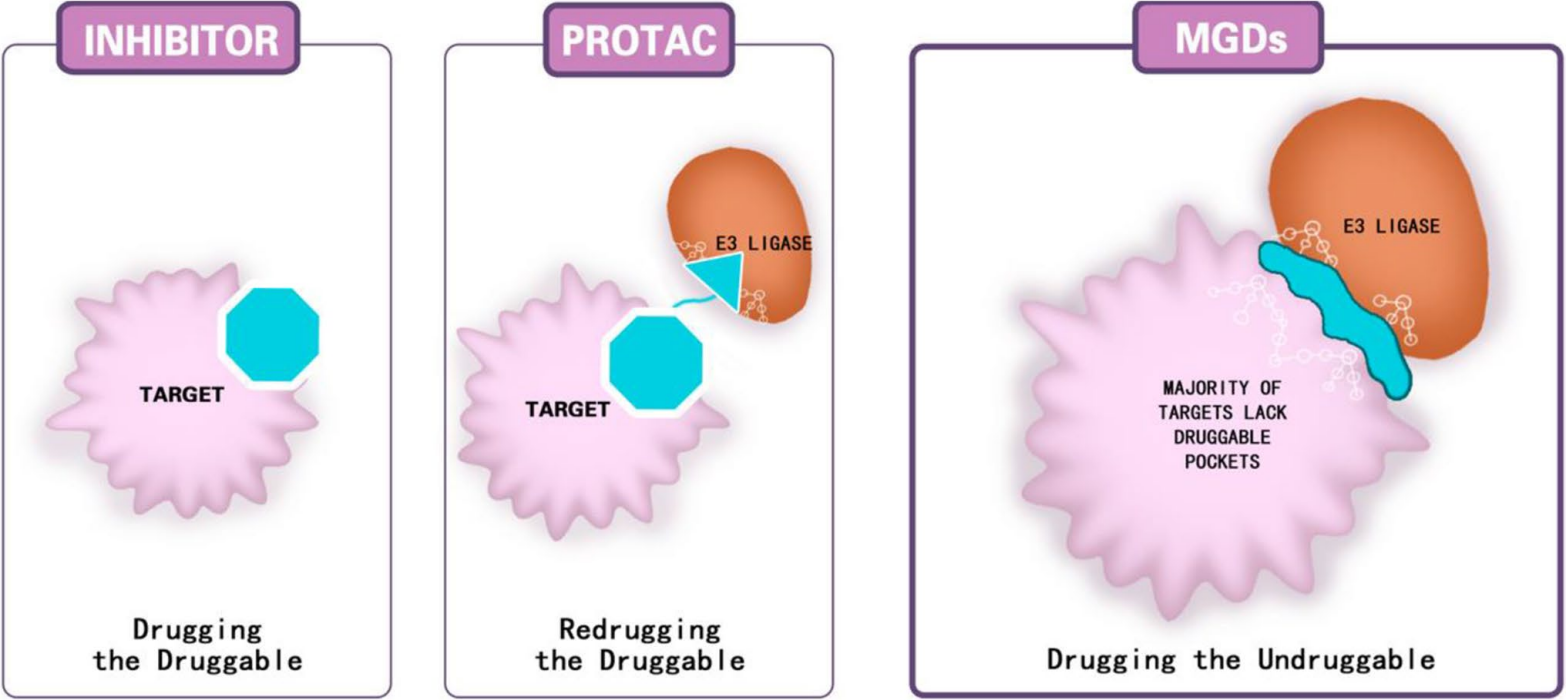
ER inhibition by ER antagonist, PROTAC, and MGDs. Target proteins possessing the binding sockets (or degrons) to which inhibitors or PROTAC linkers can bind are druggable. Otherwise, they are considered non-druggable. ER, estrogen receptor; MGDs, molecular glue degrader; POI, protein of interest; PROTAC, proteolysis-targeting chimera; SERD, selective estrogen receptor degraders; SERM, selective estrogen receptor modulator

Like PROTAC, MGD can degrade nascent ER in the cytosol before it inserts into the membrane and degrades ESR1, as well as other proteins involved in antiestrogen resistance, such as PI3K and CD4/6. Preclinical and early clinical data suggest MGD may have a higher potency than SERDs and even ER PROTAC, as well as being less toxic [58, 59]. Figure 4 compares the differences in the mechanism of actions among ER antagonists, PROTAC, and MGDs.

### LYTAC

Another protein degradation system in the cell is the lysosome. LYTACs are bifunctional molecules that bind the extracellular domain of a target protein and a cell surface lysosome-shutting receptor in order to target the protein to the lysosome [60]. LYTACs adhere to cellular receptors that facilitate the delivery of extracellular proteins to lysosomes, leading to their selective degradation of secreted and membrane-associated proteins [61]. This process is mediated using conjugates that bind both lysosomal-shuttling receptors on the cell surface and the extracellular domains of target proteins. The lysosomal pathway for protein degradation is independent of an extracellular protease to function [60], degrading proteins with both intracellular and extracellular domains. The potential of LYTACs in degrading proteins such as ER warrants further research.

### Other targets that modulate ER degradation

In addition to proteasome and lysosome protein degradation systems, there are other molecules that modulate protein degradation in breast cancer cells. BAG−1 is a molecular chaperone that affects the protein half−life of ER, PE, and Bcl−2 [62]. We also discovered its role in the prognosis of breast cancer [63]. Overexpression of BAG−1 leads to resistance to hormonal therapy by tamoxifen [64]. BAG−1 may serve as a target to modulate the half−life of ER/PR.

## Strategies to reduce target protein production

Until now, most pharmaceutical intervention for an abnormal protein or oncogene in cancer has been focused on inhibiting their function by monoclonal antibodies or small molecule inhibitors or increasing its destruction by various protein degraders. Efforts are now underway to reduce or inhibit the production of an abnormally expressed gene product by targeting its messenger RNA (mRNA) or even DNA.

### RIBOTAC

RIBOTACs are a new class of small molecules that can bind RNAs selectively, particularly those that elaborate secondary and tertiary structures. They are a promising and innovative method to target RNAs for degradation and inhibit the production of target proteins without the limitations of oligonucleotide therapies. RNA-binding molecules were linked to a small molecule that activates RNase L which is an otherwise latent ribonuclease [65]. RNase L recruits 2ʹ-5ʹ-linked tetra-adenylate (2ʹ-5ʹ A_4_), which is produced in cells during viral infection as oligoadenylates [66]. As a result, RIBOTACs cause the RNA of interest to be degraded by an active RNase L. RIBOTACs can selectively degrade targeted RNA, including that of ER mRNA.

### SiRNA

Modifying intracellular protein concentrations can also be accomplished with nucleic acid–based agents, such as antisense oligonucleotides (ASOs), or agents that rely on RNA interference (RNAi), such as siRNA. FDA has approved two ASO therapeutics (fomirisen [67] and mipomersen [67]) that reduce the production of a particular protein production. We have successfully generated aptamer siRNA against HER2/HER3 *in vitro* experiment [68] and against EGFR/HER2/HER3 [69], though they have not been tested in clinical trials due to a lack of an efficient delivery system *in vivo*. Recent developments in CRISPR/Cas9 technology [70, 71] have enabled the possibility of modifying the genome itself to achieve gene knockout. Inducing ER degradation by SiRNA can complement current SERD and decrease the number of active proteins needed to be inhibited, as well as counteract compensatory protein overexpression that is often observed following the loss of protein function. In developing a nucleic acid–based tool into a drug candidate, there are many obstacles: instability in serum, potential immunogenicity, low bioavailability, engagement of off-target mRNA, and adverse consequences from the undesired target. [72–75]

## Discussion

There are several advantages of oral SERDs over fulvestrant. Firstly, ESR1 mutation is the most common resistance mechanism to hormonal therapy in MBC [76]. Tumors with ESR1 mutation are resistant to additional therapy with AIs, yet remain sensitive to SERDs such as fulvestrant [77]. Secondly, compared to fulvestrant, oral administration of SERDs is more convenient, allowing daily and steady suppression of ERs, avoiding the painful intramuscular injection with fulvestrant, and reducing the need for office visits, which is of great importance with the current COVID-19 pandemic. Oral administration of SERDs also adds convenience as adjuvant therapy and can be combined with the systemic treatment such as CDK 4/6 and PI3K inhibitors and other targeted therapies and even immunotherapy, all of which are under current clinical research. One may argue that even if oral SERDs do not demonstrate any superiority but equal in efficacy over fulvestrant, there is still a justification to use them in preference due to these considerations. Furthermore, emerging clinical trials of oral SERD have demonstrated that they seem to be well-tolerated.

Two oral SERDs were recently reported to have failed the primary study end points, including AMEERA-3 [42] and acelERA trials [78]. Unlike the EMERALD trial, patients enrolled in the AMEERA-3 trial had to fail CDK4/6 inhibitors and two lines of hormonal therapy. In addition, measurement of ESR1 mutation was not conducted. It would be tempting to speculate that amcenestrant could still be superior to the control arm if only patients whose tumors carrying the ESR1 mutation were enrolled. acelERA is a randomized phase II trial of giredestrant vs fulvestrant or aromatase inhibitor that failed to meet its primary endpoint of superior PFS for the study drug [79], although subgroup analysis of patients with baseline ESR1 mutations appeared to show promising effects. Giredestrant did outperform anastrozole in reducing Ki67 expression and inducing complete cell cycle arrest when used as neoadjuvant therapy for previously untreated patients from the coopERA study in postmenopausal women with hormone receptor–positive, HER2 − early breast cancer [80]. Giredestrant is currently explored as first-line therapy for advanced breast cancer and early-stage breast cancer.

Before the EMERALD trial was presented, there was a concern over the possible on-target effects of oral SERDs on the cardiac conducting system and cornea. Bradycardia was reported in earlier trials of camizestrant [81] and giredestrant (GDC-9545) [47]. QT prolongation was observed in camicestrant [81] and amcenestrant [82]. Ocular toxicity is mostly seen in camizestrant [81] and giredestrant [47]. The cardiac conducting system or cornea does not express ER. Over the past decades, these toxicities were rarely observed among patients treated with antiestrogen agents, including tamoxifen, AIs, and fulvestrant. Therefore, it is unlikely the observed ocular or cardiac toxicities with certain oral SERDs are results of on-target effects against ER. It was reassuring that no ocular or cardiac toxicities were observed in the EMERALD trial. Nevertheless, the underlying mechanism of some but not other oral SERDs causing these side effects remains under scrutiny [83].

Even though the incidence of ESR1 mutation is low in HR + breast cancer not previously exposed to endocrine therapy, it rapidly increases once the HR + tumor is under the selection of antiestrogen therapy. Tumors with ESR1 mutation are known to be resistant to additional endocrine therapy with SERMs or AIs, but sensitive to SERDs such as fulvestrant. Importantly, those without ESR1 mutation may still respond to additional endocrine therapy. Sequential endocrine therapy with non-steroidal AIs such as letrozole or anastrozole followed by steroidal AI such as exemestane was widely used in the management of HR + MBC before the arrival of SERD fulvestrant and other targeted therapies such as CDK4/6 and PI3K inhibitors. With the increasing availability of genomic sequencing and escalating oncology drug cost nowadays, we strongly recommend to test all tumors for ESR1 mutation after prior AIs and CDK4/6 inhibitors or even upfront in the first-line treatment of ER + MBC, as patients carrying the mutation can be offered SERDs while those without remain as candidates for additional more affordable AIs.

In the SoFEA trial, ESR1 mutations were found in 39.1% of patients. Patients with ESR1 mutations had improved PFS after taking fulvestrant compared with exemestane, whereas patients with wild-type ESR1 had similar PFS after receiving either treatment [84]. It is clear from the EMERALD trial that patients with a tumor harboring ESR1 mutation benefitted more from elacestrant vs those without the mutation. However, elacestrant resulted in a statistically significant prolonged PFS in the overall population. It is unknown if the eventual approval of the drug would come with a companion test for ESR1 mutation and the drug is to be used only in those with ESR1 mutation or in the untested population on progression on prior AIs and CDK4/6 inhibitors. Testing of ESR1 mutation after progression on prior AIs is not the current SOC. Unlike the EMERALD (elacestrant) trial, AMEERA-3 (amcenestrant) and acelERA (giredestrant) trials did not test for ESR1 mutations. Oral SERDs such as amcenestrant were shown to be equally active against both the mutant and wild-type ER. Whether there is an underlying difference in ESR1 mutation prevalence among these three trials contributing to the observed efficacy of the three oral SERDs remains unknown. For the ongoing registration SERENA-2 trial of camizestrant, measurement of ESR1 mutants was not required. It remains guarded if any trial of oral SERDs without enrichment of ESR1 mutation will yield superior efficacy in ongoing clinical trials.

Currently, oral SERDs are being studied in different lines of therapy in HR + /HER2 − MBC and in adjuvant and neoadjuvant settings to compare their efficacy and toxicities with fulvestrant or aromatase inhibitors alone or in combination with other targeted therapies such as CDK4/6 or PI3K inhibitors. For the second-line treatment, oral SERDs are being tested against fulvestrant alone in tumors that have progressed on prior CDK4/6 inhibitors plus AIs, as in the ongoing trials with oral SERDs as in completed EMERALD trial [85] (elacestrant) or against physicians’ choice as in EMBER-3 [86] (LY3484356). For tumors that harbor PI3K mutations, oral SERDs are being investigated together with alpelicib as with the oral SERDs such as LSZ 102 [83]. For tumors that are hormonal therapy naïve or first-line therapy, oral SERDs plus CDK4/6 inhibitors are being compared with AIs plus CDK4/6 inhibitors as in AMEERA-5 [87] (amcenestrant), SERENA 4 [88](camizestrant), or with physicians’ choice plus CDK4/6 inhibitors as in acelERA Breast Cancer [78] (giredestrant) and in tumors with ESR1 mutation, as in SERENA 6 [89] (camizestrant). In the neoadjuvant setting, oral SERDs are being explored in locally advanced HR + /HER2 − stage III breast cancer against AIs alone as in AMEERA-4 [90] (amcenestrant) or in combination with CDK4/6 inhibitors as in coopERA BC study [80](giredestrant). Similarly, in the adjuvant treatment of early breast cancer, oral SERDs are being studied against AIs or physicians’ choice as in the Lidera Breast Cancer Study [91] (giredestrant) or tamoxifen as in AMEERA-6 [92] (amcenestrant) in HR + /HER2 − stages I–II early breast cancers. Oral SERDs are likely to replace fulvestrant in the metastatic setting for second-line therapy due to its oral formulation and lack of local injection reaction, even if the randomized clinical trials do not demonstrate superior but non-inferior efficacy. For the first-line treatment of HR + /HER2 − MBC and in neoadjuvant and adjuvant setting, oral SERDs have the potential to replace AIs or tamoxifen due to their activity against tumors bearing ESR1 mutation that causes primary resistance to AIs or tamoxifen.

In addition to oral SERDs, a variety of protein degradation technologies are under development, aiming to inhibit the ER pathways in ER + breast cancer. ER degradation by SERDs relies on recognition by E3 ligase and the proteasome system, a rather “passive” process. Denatured ERs are among other obsolete proteins to be degraded by the E3-proteasome system. On the other hand, the degradation by PROTAC against ER is “active” using a bispecific linker that links the E3 ligase and the ER to recruit the E3-proteasome system specifically. Therefore, there is a theoretical advantage of PROTAC-ER degrader over the other SERDs in terms of the degree of ER degradation. Preliminary research showed that ARV-471, an oral PROTAC degrader, achieved a higher degree of ER degradation compared to fulvestrant. The initial clinical trial in breast cancer demonstrated encouraging results. In 60 patients who failed multiple therapies, AVR-471 resulted in a clinical benefit rate (CBR) of 40%, and three patients showed confirmed PR. More importantly, from the paired biopsy, ARV-471 reduced in ER at all dose levels, reaching a 90% reduction of ER at the highest level.

While PROTAC can actively degrade the target protein, the technology requires the presence of degron, the binding site for the PROTAC linker. Many target proteins may not have an identifiable degron and are therefore non-druggable. The MGD does not require such degrons, making it possible to drug the undruggable proteins. Whether it is more effective than PROTAC in protein degradation, including that of ER, is currently unknown.

While PROTAC and MGD may degrade intracellular protein targets, LYTACs can effectively target membrane-bound and even secreted protein [60, 93]. An individual LYTAC’s success is likely to be determined by a variety of factors, such as the endogenous trafficking mechanisms of proteins, the amount of surface localization, and the intrinsic susceptibility to lysosomal transport via clathrin-mediated endocytosis, as well as the stoichiometry relative to the LYTAC to the receptor. Pharmacokinetics must be controlled to reduce the off-target clearance and stoichiometries of LYTACs to degrade a membrane protein effectively [60]. Due to their chemical tunability and modularity, LYTACS will provide new opportunities to target protein degradation of secreted and membrane proteins for both research and potential therapeutic purposes such as ER.

Finally, as in medicine, the ultimate alteration of a protein function is not through inhibition or destruction, but through prevention, i.e., the elimination of protein production. This can be achieved by newer technologies such as RYBOTAC and siRNA or aptamers, even though these approaches still need to overcome many obstacles to reach the clinical development stage, including the effective method of *in vivo* delivery to the tumor targets.

RIBOTAC, as a small molecule, might have better pharmacokinetics compared with oligonucleotide-based treatment. Animal studies suggested its broader tissue distribution [94]. The catalytic nature makes RIBOTACs degrade RNA at a lower concentration. However, it is more challenging to develop RIBOTACs than an oligonucleotide-based treatment due to RIBOTAC being a small molecule with high functional selectivity. In contrast, antisense oligonucleotides can be designed with a complementary RNA sequence to bind to specific targeted RNA, despite variable specificities. siRNA has the potential to target specific mRNA of interest to inhibit protein production such as ER. In developing a nucleic acid–based tool into a drug candidate, there are many obstacles to overcome, including their instability in serum, potential immunogenicity, low bioavailability, engagement of off-target mRNA, and adverse consequences from the undesired target [72–75]. The COVID-19 pandemic saw a rapid expansion of mRNA-based therapy in medicine with the successful development of a delivery system to produce the COVID-19 mRNA vaccine. A similar delivery system is being explored to deliver siRNA and even aptamers to target biomarkers such as ER or oncogenes in oncogenesis and tumor progression [95].

## Conclusion

Endocrine therapy is the most important treatment for ER + breast cancer. An important escape mechanism to endocrine therapy is the development of ESR1 mutation, which occurs frequently in tumors previously exposed to SERM or AIs. While the ESR-bearing breast cancers are resistant to additional AIs, they remain sensitive to SERD such as fulvestrant. Clinical application of fulvestrant is limited by its intramuscular formulation, once-monthly injection, and incomplete ER degradation. Oral SERDs are emerging as a potential replacement of fulvestrant in breast cancer, with many in phase III clinical trials. Elacestrant is the first oral SERD that completed the phase III trial showing superior efficacy, especially in tumors harboring ESR1 mutation and good tolerability, without unexpected on-target toxicities. While other oral SERDs did not meet their trial endpoints, the lack of enrichment of tumors with ESR1 mutation may partly explain the negative results. Testing for ESR1 mutation in ongoing oral SERD trials and their clinical application is highly recommended. Other novel protein degradation technologies such as PROTCs, LYTACs, MGD, RIBOTAC, and siRNA may result in a higher degree of ER degradation or inhibit ER production. Their potentials in the hormonal treatment of breast cancer are being actively explored.
